# Effect of Methyl Jasmonate Doped Nanoparticles on Nitrogen Composition of Monastrell Grapes and Wines

**DOI:** 10.3390/biom11111631

**Published:** 2021-11-04

**Authors:** Rocío Gil-Muñoz, María José Giménez-Bañón, Juan Daniel Moreno-Olivares, Diego Fernando Paladines-Quezada, Juan Antonio Bleda-Sánchez, José Ignacio Fernández-Fernández, Belén Parra-Torrejón, Gloria Belén Ramírez-Rodríguez, José Manuel Delgado-López

**Affiliations:** 1 Murcian Institute of Agricultural and Environment Research and Development, Calle Mayor s/n, 30150 La Alberca, Spain; mariaj.gimenez8@carm.es (M.J.G.-B.); juand.moreno5@carm.es (J.D.M.-O.); diegof.paladines@carm.es (D.F.P.-Q.); juanantonio.bleda@carm.es (J.A.B.-S.); josei.fernandez@carm.es (J.I.F.-F.); 2 Department of Inorganic Chemistry, Faculty of Science, University of Granada, 18071 Granada, Spain; belenparrato@ugr.es (B.P.-T.); gloria@ugr.es (G.B.R.-R.); jmdl@ugr.es (J.M.D.-L.)

**Keywords:** amino acids, ammonium, elicitors, nanotechnology, *Vitis vinifera*

## Abstract

Nitrogen composition on grapevines has a direct effect on the quality of wines since it contributes to develop certain volatile compounds and assists in the correct kinetics of alcoholic fermentation. Several strategies can be used to ensure nitrogen content in grapes and one of them could be the use of elicitors such as methyl jasmonate. The use of this elicitor has been proven to be efficient in the production of secondary metabolites which increases the quality of wines, but its use also has some drawbacks such as its low water solubility, high volatility, and its expensive cost. This study observes the impact on the amino acid and ammonium composition of must and wine of Monastrell grapes that have been treated with methyl jasmonate (MeJ) and methyl jasmonate n-doped calcium phosphate nanoparticles (MeJ-ACP). The first objective of this study was to compare the effect of these treatments to determine if the nitrogenous composition of the berries and wines increased. The second aim was to determine if the nanoparticle treatments showed similar effects to conventional treatments so that the ones which are more efficient and sustainable from an agricultural point of view can be selected. The results showed how both treatments increased amino acid composition in grapes and wines during two consecutive seasons and as well as the use of MeJ-ACP showed better results compared to MeJ despite using less quantity (1 mM compared to 10 mM typically). So, this application form of MeJ could be used as an alternative in order to carry out a more efficient and sustainable agriculture.

## 1. Introduction

The nitrogen statues of the grapevine (protein, peptides, and amino acids) can influence the concentration and composition of quality components in the grape berry and therefore in the wines. In musts, nitrogen composition plays a key role on wine quality because certain amino acids are precursor of important fermentative volatile compounds, mainly of higher alcohols and ethyl esters, which contribute to the pleasant aroma of wine [1,2]. In addition, must nitrogen content can affect the dynamic and the development of alcoholic fermentation [3]. Stuck and sluggish fermentations are one of the major enological problems resulting in increased vinification time and spoilage of wine [4] due to a lack of nitrogen in the must. Contrarily, a high nitrogen concentration in grapes could lead to the synthesis of biogenic amines in musts and wines, which can cause adverse health troubles to the consumers [5].

It should be noted that *Saccharomyces cerevisiae* does not consume nitrogen randomly but has developed different molecular mechanisms to select preferred nitrogen sources in the first place. Yeasts use a mechanism called nitrogen catabolite repression (NCR), which mediates the selection of good nitrogen sources by the expression of appropriate transport systems (permeases) and the degradation of non-appropriate permeases [6,7]. In this sense, ammonium ions and free amino acids are easily assimilable by yeasts. Hence, the usable nitrogen fraction corresponds to the sum of ammonium ions and amino acids, except proline, and it is referred to as yeast assimilable nitrogen (YAN). A concentration ranged 70–267 mg N/L or YAN is generally an accepted level for red musts concentration for the completion of fermentation in under normal sugar concentrations, although in past was considered 140 mg N/L of YAN a practical minimal limit [1,8].

Nitrogen content and amino acid profile in grapes vary depending on the nature of soil, cultivar, rootstock, nitrogen fertilization, vineyard health, maturation stages or cultivation systems [1,3,9,10]. This content can be manipulated in grapes using viticulture strategies; and one of them is the use of elicitors. Amongst elicitors, methyl jasmonate (MeJ) is an organic volatile compound derived from jasmonic acid that is present in several plant tissues and has a key role as a signal molecule and inductor of secondary metabolites in plants [11]. Several studies have shown how MeJA application enhances anthocyanin accumulation in soybean seedlings [12] peach shoots [13] apple fruit [14] pomegranates [15], blueberries [16] grapes [17], and strawberries [18] or is able to stimulate accumulation of stilbene in leaves and berries of grapevine plants [19]. It has also been reported that MeJ foliar applications induce an increase or decrease of some amino acids. In this way, Garde-Cerdán et al. [20] showed an increase in content of histidine (His), serine (Ser), tryptophan (Trp), tyrosine (Tyr), asparagine (Asn), methionine (Met) and lysine (Lys) in the must from Tempranillo grapes using MeJ, while Gutiérrez-Gamboa et al. [21] reported a decrease in the concentration of several amino acids after the use of different elicitors in Cabernet Sauvignon grapes. 

In general, despite the good results obtained in grapes with this elicitor, some disadvantages have been observed in the use of foliar MeJ, such as its low water solubility, high volatility and its expensive cost. Several studies have reported that the increase of grape and wine polyphenols using foliar applications of MeJ in leaves, requires high concentrations of MeJ, such as Ruiz-García et al. [22,23]; Gil-Muñoz et al. [24] or Portu et al. [25] in which 10 mM MeJ concentrations were used to increases secondary metabolites in Monastrell or Tempranillo grapevine varieties.

In recent years, nanotechnology has presented a significant potential as an innovative tool for the safer and more efficient delivery of agrochemicals [26,27]. Recent approaches have incorporated nano-enabled products to treat nutrient disorders, enhance crop productivity and reduce nutrient loss in crops [28,29]. To the best of the authors’ knowledge, only very few studies have been performed incorporating nanoparticles (NPs) to crop stress management. The use of nanoparticles for targeted delivery of elicitors in plants could minimize losses and enhance the absorption on behalf of the plant. Several nanoparticle types are starting to be used in agriculture, but the use of calcium phosphate nanoparticles in crops, in order to use less pesticides and increases their quality, has received much less attention. Very recently, we developed nanoelicitors which consisted of amorphous calcium phosphate nanoparticles (mimicking the precursor phase of bone mineral) [30]. We demonstrated that this type of nanoparticles can retain and protect MeJ on the surface of the leaves during a longer period of time. This protective action along with the slow and gradual release over time provided a sustained and prolonged delivery of the resistant-inductor elicitor, resulting in a much higher efficiency [31]. However, no study has dealt with the impact of methyl jasmonate n-doped calcium phosphate nanoparticles (MeJ-ACP) on the accumulation of nitrogen in Monastrell grapes and wines.

For this reason, the purpose of this paper was to provide information on the effect of elicitation with MeJ in a conventional manner and MeJ supported on calcium phosphate nanoparticles and compare the results on grape and wine amino composition in order to show another alternative which is more sustainable and cleaner in vineyards.

## 2. Materials and Methods

### 2.1. Experimental Design in Field and Treatments

The study was performed on two consecutive vintages (2019 and 2020) on 14-year-old *Vitis vinifera* L. cv Monastrell vineyards grafted onto 1103-Paulsen (clone 249) rootstock and trained in a bilateral cordon training system trellised to a three wire was used. Vine rows were arranged N-NW to S-SE with between-row and within-row spacing of 3 × 1.25 m and located in Cehegín (Murcia, Spain- latitude 38°6′39.35″, length 1°40′59.06″ and altitude of 433 m). The vineyard was drip-irrigated and liquid organic matter was added as a fertilizer. The experiments were conducted in a randomized block design, in which all treatments were applied to three replicates, using 10 vines for each replication. Two foliar treatments were applied to the plants in spray form as a water suspension of MeJ 10 mM (methyl jasmonate) (Sigma Aldrich, St. Louis, MO, USA) and a water suspension of MeJ-ACP 1 mM (Mej-doped calcium phosphate nanoparticles) at veraison. Approximately 200 mL of the product was applied to each plant prepared with Tween 80 (Sigma Aldrich, St. Louis, MO, USA) as the wetting agent (0.1% *v*/*v*). Control plants were sprayed with aqueous solution of Tween 80 alone. For all treatments, a second application was performed 7 days after the first.

### 2.2. Synthesis of MeJ-ACP

The synthesis of MeJ-ACP nanoparticles was carried out through a simple and eco-friendly method previously reported [30]. Briefly, it consisted of mixing 2 L of an aqueous solution (i) containing 0.2 mol L^−1^ Ca (NO_3_)_2_, 0.2 mol L^−1^ sodium citrate (Na_3_Cit) with an equal volume of solution (ii) containing 0.12 mol L^−1^ K_2_HPO_4_ and 0.1 mol L^−1^ Na_2_CO_3_. The mixture was then kept at 37 °C for 5 min. After that, the precipitate was repeatedly washed with ultrapure water by centrifugation (3700 rpm, 15 min, 4 °C). The collected ACP nanoparticles were dispersed in 2 L of ultrapure water with vigorous vortex and then, 5 mL of MeJ were added to the nanoparticle suspension. After 24 h under agitation, MeJ-ACP nanoparticles were collected by centrifugation (3700 rpm, 15 min, 4 °C). MeJ loading was estimated by UV-Vis spectroscopy, as previously described [30]. Nanoparticles contained 6% *w*/*w* of MeJ. 

### 2.3. Climatological Conditions

Climatic information related to radiation (w/m^2^); rainfall (mm) and seasonal temperatures (°C), for each season was recorded by the Agricultural Information System of the Region of Murcia (SIAM), located 50 m from the experimental field. This information is shown in Table 1.

### 2.4. Physicochemical Parameters of Grapes at Harvest

Total soluble solids, pH, total acidity, tartaric acid and malic acid were determined in grapes at harvest. Harvest time was determined when the technological maturity was reached in grapes. Total soluble sugars were measured using an Abbé-type refractometer (Atago RX-5000, Tokyo, Japan) and pH and total acidity using and automatic titrator (Metrohm, Herisau, Switzerland) with 0.1 N NaOH. Tartaric and malic acid were measured using enzymatic kits from Boehringer 146 Mannheim GmbH (Mannhein, Germany). The methodology used to carry out these analyses is described in OIV (Organisation Internationale de la Vigne et du Vin) [32].

### 2.5. Vinifications

The grapes were harvested manually after reaching their optimum moment of ripening (technological maturity). Vinifications were conducted in triplicate for each of the treatments following a traditional methodology how it is explained below. The grapes were destemmed, crushed and sulphited (8 g SO_2_/100 kg grapes) and subsequently introduced into the tanks (50 L). Total acidity was corrected to 5.5 g/L with tartaric acid and selected yeasts (25 g/HL of yeasts Zymaflore FX10-Affort, Gipuzkoa, Spain) were inoculated. Throughout the maceration period (8 days), the cap was punched down twice a day, and the temperature and density were recorded. At the end of this period, the wines were pressed at 1.5 bars in a 75 L tank membrane press. Free-run and press wines were combined and stored at room temperature. When reducing sugars measured <0.2 g L^−1^ and the density was <1.0 g mL^−1^, the alcoholic fermentation was terminated. The analyses were carried out at the end of the alcoholic fermentation in triplicate.

### 2.6. Pre-Sample Preparation for Analysing Nitrogen Composition in Grapes and Wines

Amino acid composition, ammonium and YAN were performed by the method described by Gómez-Alonso et al. [33] with some modifications. Briefly, 40 berries of each sample were hand crushed and centrifuged for 15 min at 4500 rpm (J-P Selecta, S.A., Barcelona, Spain). The supernatant was recentrifuged in an Eppendorf model 5810-R centrifuge (Thermo Fisher Scientific, Madrid, Spain) for 5 min at 10,000 rpm. The resulting supernatant derivatization process was carried out by mixing 1.750 mL of borate buffer (obtained from a mixture of boric acid with a solution of NaOH at 40%), 750 μL of MeOH, 1 mL of supernatant obtained from each of the samples to be analyzed (or 1 mL of previously filtered wine), 20 μL of internal standard DL-2-Aminoadipic acid (Sigma Aldrich, St. Louis, MO, USA) and 30 μL of diethyl-ethoxymethylenemalonate (Sigma Aldrich, Buchs, Switzerland). Subsequently, the samples were gently shaken and introduced into an ultrasound (J-P Selecta, S.A., Barcelona, Spain) for 30 min; then they were placed in a Memmert Model U40 oven (Memmert GmbH + Co. KG, Schwabach, Germany) at a temperature of 75 °C for two hours. After this time, the samples were cooled. 

### 2.7. Determination of Nitrogen Composition by HPLC

The HPLC analysis was performed on a Waters 2695 liquid chromatograph (Waters Corporation, Milford, MA, USA) equipped with a Waters996 diode array, and a Cortecs Shield RP18 column (Crawford Scientific, Strathaven, UK), 4.6 × 150 mm, 2.7 µm particle size were used to analyze the musts and wines. These analyses used solvents acetate buffer (A) and MeOH-Acn (20:80) at a flow of 0.6 mL min^−1^. The compounds were identified by comparing their retention times and their UV-visible spectra with those of the corresponding standards (Sigma-Aldrich, Madrid, Spain). The ammonium ion (NH_4_^+^) and the following free amino acids were quantified using the corresponding calibrated curves: aspartic acid (Asp), glutamic acid (Glu), serine (Ser), asparagine (Asp), glutamine (Gln), histidine (His), glycine (Gly), threonine (Thr), β-alanine (β-Ala), arginine (Arg), α-alanine (α-Ala), γ-aminobutyric acid (GABA), proline (Pro), tyrosine (Tyr), valine (Val), methionine (Met), cysteine (Cys), isoleucine (Iso), leucine (Leu), tryptophan (Trp), phenylalanine (Phe), ornithine (Orn) and lysine (Lys).

### 2.8. Statistical Analysis

The significant differences between the concentrations of each of the compounds analyzed in musts and wines were evaluated by an analysis of variance (ANOVA) and a multivariate factor analysis (MANOVA). The separation of means was carried out using Duncan’s test (*p* ≤ 0.05). A discriminant analysis was also carried out to determine whether the groups were sufficiently discriminated based on the original variables available, obtaining a clear discrimination between treatments and control samples. The performance of these analyses was carried out using the statistical package Statgraphics 5.0 Plus (Statpoint Inc., Warrenton, VA, USA).

## 3. Results and Discussion

### 3.1. Must Enological Parameters

The physicochemical data of treated (with MeJ and with MeJ-ACP) and untreated Monastrell must are presented in Table 2. Five parameters were evaluated at harvest time during two seasons: total soluble solids (as °Brix), total acidity, pH, tartaric and malic acid.

The results obtained did not show statistically significant differences between the different treatments and the control grapes during 2019. Similar results were reported by other studies with MeJ which did not show significant differences in the physicochemical parameters of the must of treated vines compared to untreated vines in the Tempranillo variety [20,34]. However, during the following season, it could be observed how the application of MeJ significantly increased the total acidity and the levels of malic acid. Similar results were also found by Gil-Muñoz et al. [24] in Monastrell grapes treated with MeJ and BTH (benzothiadiazole) and by Ruiz-García et al. [22] who showed a slight increase in total acidity in grapes treated with MeJ and BTH. However, regarding total soluble solids, other studies have reported a decrease in the °Brix in grapes treated with MeJ, such as Paladines-Quezada et al. [35] in Monastrell or D’Onofrio et al. [36] in Sangiovese grapes. These results could show the relationship between climatic conditions and the high response to treatments when rainfall was scarcer (Table 1).

### 3.2. Must Amino Acids and Ammonium Content in Grapes

Table 3 shows amino acids and ammonium concentration (mg N L^−1^) results obtained in musts from non-treated and treated Monastrell grapes with MeJ and MeJ n-doped calcium phosphate nanoparticles (MeJ-ACP) during two seasons.

In general, the most abundant amino acids found in control grapes were Pro, Arg + GABA, Glu, Asp + Ser and Glc in 2019, representing around 67 % of total amino acids (Table 3), while the least abundant amino acids were Orn and Lys, accounting for 0.8% of total amino acids. In 2020, the most abundant amino acids found in control grapes were Pro, Arg + GABA, Glc, Asn + Ser and Trp and the less abundant ones were again Orn and Lys. One study on Monastrell grapes showed that Asp, His, Arg, and Ala were the most abundant amino acids [37] and in other study, Gln, Arg, Ala, GABA and Pro were the most abundant amino acids in this variety [38]. The most abundant amino acids found in grape juice are usually Glu, Pro and Arg, while generally the least abundant amino acids found in grape juice are Met, Cit and Cys [39]. On the other hand, YAN values obtained during the two years and for all the treatments were much higher than the stipulated limits for a good development of the alcoholic fermentation (range between 354–1195 mg N L^−1^). A concentration ranged 70–267 mg N/L or YAN is generally an accepted level for red musts concentration for the completion of fermentation under normal sugar concentrations, although in the past 140 mg N/L of YAN was considered a practical minimal limit [1,8].

In our study, foliar treatment applications favored total amino acids, YAN (total amino acids without Pro) and ammonium content in musts even though some differences were found between seasons and treatments, although these differences were more noticeable in 2019 compared to 2020 (Table 3). These results could be attributed to the climatic differences between the two years; 2020 was a drier year with slightly higher temperatures than 2019. Bouzas-Cid et al. [40] reported that season was a dominant variability factor of the concentration of Asp, Ser, His, Thr, Arg, Tyr, Val, Met, Cys, Ile, Trp, Leu and Phe, after irrigation treatments were performed to Treixadura grapevines in a study carried out during three seasons. Likewise, Gutierrez-Gamboa et al. [41] also found variability between seasons in a study performed in Tempranillo Blanco treated with seaweed. In addition, the positively significant influence on the concentration of amino acids and ammonium of the different treatments with MeJ can be explained by the ability of MeJ to induce the production of metabolites by the plant [42].

Regarding the effect of the different treatments applied to the grapevines in 2019, it was observed that the MeJ, in a conventional form or as nanoparticles, had a significant effect on total must amino acid concentration, being positively affected by the application of both treatments compared to the control samples (Table 3). In 2019, the treatment with MeJ was the one that presented a greater increase in the total concentration of amino acids, also showing a statistically significant increase in each of the individual amino acids compared to the control. However, the application of MeJ-ACP did not show significant differences in Ala, Tyr and Met. During this season, MeJ application to grapes increased the concentration of the all amino acids analyzed excepted for Cys; and MeJ-ACP applications increased the concentration of all amino acids except in α-Ala, Tyr and Met. Ammonium was also increased by both treatments. Contrarily to this, Gutierrez-Gamboa et al. [43] reported that MeJ applications in Garnacha decreased the content in must of eighteen amino acids compared to control grapes. The most important yeast nitrogen sources among the amino acids are Asp, Glu, Asn, Gln, His, Thr, Arg, Ala, and Tyr [44]. Therefore, our study observes how foliar Mej but also MeJ-ACP applications were able to increase some of the amino acid precursors (Thr, Val, Iso, Leu, Trp and Phe) for fermentative volatile compounds. By contrast, other amino acid precursors like Met or Cys, precursors of volatile sulfur compounds, such as methanethiol and hydrogen sulfide, which contribute to rotten eggs, cooked cabbage, and onion flavor notes to the wines [45]; were not increased in MeJ-ACP treated grapes. It is very remarkable that the results obtained with MeJ-ACP were using 10-fold less concentration of MeJ than in conventional form, so it would be a good alternative to increase the nitrogen compounds in grapes using a cleaner agricultural strategy.

During 2020 musts from grapes treated with MeJ presented a higher concentration of Glu, Asn + Ser, Gln, His, β-Ala, Arg + GABA, α-Ala, Pro, Tyr, Trp, Orn and Lys than control grapes. When the grapes were treated with MeJ-ACP, they also increased their concentration in the following amino acids: Glu, Asn + Ser, His, β-Ala, Arg + GABA, α-Ala, Pro, Tyr, Met, and Iso. As occurred in 2019, ammonium content in must also was increased with both treatments compared to control musts. In this way, a concentration ten times lower in the form of nanoparticles, was able to increase the concentration of all these amino acids together with the ammonium. Different results have been reported regarding the effects of MeJ on amino acid composition in Tempranillo grapes, Garde-Cerdán et al. [20] found an increase in His, Ser, Trp, Phe, Tyr, Asp, Met and Lys compared to control grapes, but in other study made by Gutierrez-Gamboa et al. [46] they only found an increase in Met and Phe.

In addition, Table 3 shows a multifactorial analysis performed by each amino acid and ammonium according to treatment, season, and their interaction (Table S2 indicates the percentage of variance of each factor and its interaction). The treatment was a dominant factor of variation for all amino acids, ammonium, total amino acids without Pro and total amino acids. Season was the dominant factor of variation for all amino acids, including total amino acids without Pro and total amino acids, except for Val and ammonium. The interaction between treatment and season factors was dominant in the variation of all amino acids (except His and Cys), ammonium, total amino acids and total amino acids without Pro. Certain authors found links between the must amino acid composition and volatile compounds observed in wines [47,48].

### 3.3. Proline/Arginine Relationship

Pro to Arg ratio is commonly used to classify grape varieties according to their ability to accumulate either one or the other of these two amino acids [1,42,49]. Proline and arginine are the most abundant amino acids in grapes and play an important role during alcoholic fermentation. Arginine is the first amino acid consumed by yeasts, while proline is relatively non-assimilable under anaerobic fermentation conditions [50].

In our study (Table 3), the Pro/Arg ratio obtained in control samples was relatively high overall in 2019 (4.7), confirming that Monastrell variety is an accumulating variety of Pro. This statement coincides with other studies previously conducted by Garde-Cerdán et al. [38] in Monastrell vineyards. As well, Gutierrez-Gamboa et al. [37] showed in a study with Carignan noir or Tempranillo that they were also varieties that are “proline accumulators”. Therefore, low total N and high proline concentrations can result in low yeast biomass and stuck or sluggish fermentations [51].

Regarding treatments, although there are hardly any differences in the Pro/Arg ratio between the berries treated with MeJ (≈2) and those treated with MeJ-ACP, it is shown that the use of MeJ and MeJ-ACP as elicitors increases the percentage of nitrogen easily assimilated by the yeasts in Monastrell. Again, it is remarkable to observe in the results the use of tenfold less MeJ concentration when we used MeJ-ACP. In other varieties such as Tempranillo [18] the Pro/Arg ratio was not affected by the use of MeJ. Therefore, according to our results, Monastrell behaved as a proline accumulator variety, but the treatments applied were able to dismiss the ratio proline/arginine converting its grapes into greater assimilable nitrogen accumulators as arginine. Varieties such as Gewürtztraminer and Muscat or Tempranillo Blanco [20] are also high arginine-accumulating cultivars. However, in contrast, the arginine concentration in Cabernet Sauvignon and Chardonnay berries is lower because they are cultivars with high proline accumulation [38].

### 3.4. Amino Acids and Ammonium Content in Wines

Table 4 shows amino acids and ammonium results obtained in wines from Monastrell grapes nontreated and treated with MeJ and MeJ-doped-nano (MeJ-ACP) during two seasons (2019 and 2020).

The most abundant amino acid found in Monastrell wines was Pro in both seasons, accounting for 73% (mean) in 2019 and 84% in 2020. By contrast, Oliva et al. [37] found in a study with Monastrell grapes located in Jumilla (Spain) that Arg and Ala were the most abundant amino acids, representing about 50% of total amino acids. According to Gutierrez-Gamboa et al. [39], the most abundant amino acids observed in red wines are usually Pro, Arg, Glu, Ala and Asp, while the least abundant amino acids generally found in these wines are Cys, Tyr, Ile, Trp and Leu. Wines with higher amounts of residual nitrogen together with other factors, have greater risks of microbiological instability and the production of ethyl carbamate and biogenic amines [52,53,54,55,56]. As it has been mentioned before, yeast assimilable nitrogen (YAN) is a combination of free amino acids and ammonium (NH_4_), both the main nitrogen sources since they contribute to the sensory properties, microbiological stability and flavor of wines [57,58] and also proline. 

During 2019, wines from the grapes treated with MeJ showed a higher concentration of Asp, Glu, Asn + Ser, Glc, His, Gly, Thr, β-Ala, Pro, Tyr, Iso, Leu, Trp, PHe, Or, Lys than control wines. Additionally, MeJ-ACP wines, also showed higher concentrations of all previous mentioned amino acids compared to control wines, excluding Iso and Lys. Ammonium levels were 14.9 mg L^−1^ for control grapes and 23.9 and 33.0 mg L^−1^ for MeJ-ACP and MeJ respectively. The range of this cation is between 19 and 240 mg L^−1^ in in Californian grapes [59], between 43–115 mg L^−1^ in Stellenbosch varieties [60] or 17–156 mg L^−1^ in French grapes [8]. The concentration of several amino acids changes throughout the winemaking process due to Arg, Gln, Thr and Trp being consumed by yeast during alcoholic fermentations [41]. On the other hand, Pro widely increases its concentration throughout the vinification process, being one of the most released amino acids by the yeast by the end of alcoholic fermentation, probably associated with yeast autolysis [3].

During 2020, the differences between control and treated wines were less evident, as also occurred in musts, but wines from grapes treated with MeJ showed higher concentrations in Asp, Glu, Asn + Ser, Gln, Gly, Thr, Arg, GABA, α-Ala, Pro, Tyr, Val, Leu, Thr, Phe, Orn, and Lys, and ammonium than control wines. By contrast, the wines from grapes treated with MeJ-ACP, these differences were only found in the following amino acids: Asp, Gln, Pro and Thr, demonstrating how the foliar application of nitrogen sources did not affect the amino acid composition of Tempranillo wines [59]. However, it is noticeable that during this season, wines from grapes treated with MeJ-ACP did not have more of certain amino acids (His, Tyr, Lys, Phe, and Arg) which are known to be precursors of biogenic amines (histamine, tyramine, cadaverine, phenylethylamine and putrescine) which are undesired in wine production due to their negative health effects and unpleasant odors [56,57]. 

These results were obtained at the end of alcoholic fermentation but Alcaide-Hidalgo et al. [61] studied the influence of malolactic fermentation, postfermentative treatments and aging with lees, on nitrogen compounds in Tempranillo wines, showing the disappearance of Arg and Tyr and the formation of Orn, as unique changes. Therefore, it is to be expected that in the evolution of these wines there will be no appreciable changes in their nitrogen composition over time.

In addition, Table 4 shows multifactorial analysis performed by each amino acid, ammonium, total amino acids and total amino acids without Pro, according to the treatment, season, and their interaction (Table 3 indicates the percentage of variance of each factor and its interaction). Treatment was dominant for all amino acids except for β-ala and Cys, ammonium, total amino acids and total amino acids without Pro. However, season affected all the measured parameters. The interaction between treatment and season was a dominant factor of variation of the concentration of Thr, Arg, α-ala, Tyr, Leu, Trp, Phe, Orn, total amino acids without Pro, and ammonium.

The multifactorial analysis showed that the type of treatment is a relevant variation factor both for the total amino acids and for each amino acid in particular except for β-Ala and Cys for which it did not present significant differences between treatments. This could be due to an optimal consumption of these amino acids by the yeasts, matching the concentration of the treated wines to the control wines. The vintage also represented an important variation factor for all amino acids, which presented very significant differences between the different years except for Met, which, although it showed statistically significant differences, were not as noticeable as in the rest of the amino acids. Between vintages differences were due to climatic conditions (Table 1) and the difference in the maturation of the berries in their collection (Table 2). However, the interaction between treatment and year did not present statistically significant differences for most of the amino acids except for Thr, Arg, α-Ala, NH_4_^+^, Tyr, Leu, Trp and Phe.

Finally, Hernández-Orte et al. [44] reported that aspartic acid, alanine, phenylalanine and threonine added to the must have a considerable influence on the wine aroma compounds obtained in the fermentation process, decreasing sulfur notes and increasing floral notes considerably. In our case, the treatments increased these parameters in the wines for both years, provided the grapes were treated with MeJ, and for MeJ-ACP, except for Thr and Phe during 2020.

### 3.5. Discriminant Analysis

**Figure 1:**
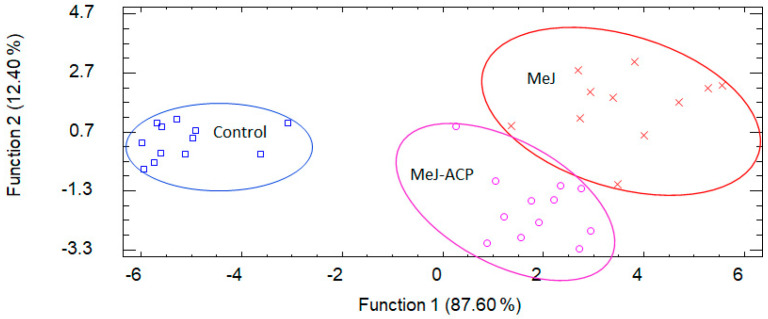
Distribution of samples (grapes and wines) in the coordinate system defined by the discriminant functions according to the treatments.

Several chemometric procedures have been used to differentiate wine composition in amino acids such as cluster analysis, principal components, and discriminant analysis. In our study, a discriminant analysis was carried out to check whether with the measured variables we could classify our samples according to the applied treatments. Four discriminant functions with a *p*-value less than 0.05 were calculated and therefore statistically significant with a confidence level of 95%. These four discriminant functions allowed us to 100% correctly classify the samples according to the applied treatment (Figure 1). The relative percentage for function 1 was 58.5% and 20.4% for function 2. As can be seen, the separation of the two applied treatments (MeJ and MeJ-ACP) regarding the control samples were very good (Figure 1). The two treatments were located in the central and right part of the graph, being MeJ treatment found further from the control indicating greater differences with respect to the control. The treatment carried out with the nanoparticles was located in the middle of the graph, thus resulting in intermediate values between those found in the control and the treatment with MeJ. It is logical since when we applied this treatment, we were using tenfold less concentration of MeJ than in conventional form. Nevertheless, MeJ-ACP treatment prompted to relevant differences from the control samples.

Table 5 shows the standardized coefficients of the functions used to discriminate between the different treatments. From the different magnitude of these coefficients, it can be determined how the independent variables are used to discriminate between groups. The variables with the highest discriminating power were the following amino acids for function 1: Iso, Val, Pro, Arg, GABA and Glu; and for function 2 they were Val, Leu, Asp and Ser.

## 4. Conclusions

Foliar applications of methyl jasmonate in Monastrell grapes increased the concentration of several amino acids in must and wine. The application of elicitors (MeJ and MeJ-ACP) significantly increased the nitrogen composition of the musts and wines of the Monastrell variety, although the results obtained were influenced by the climatic differences experienced during the two years of study. During the first season the differences obtained between the treatments and the control vines was more noticeable. However, the Pro/Arg ratio decreased with the treatments, thus increasing the YAN for the yeasts. This decrease was more evident in the nanoparticle treatments despite using tenfold less MeJ concentration than free MeJ application. The treatment with nanoparticles (MeJ-ACP) increased the nitrogen composition of the grapes and the wines in relation to the untreated vines, although the results were not as good as in the case of conventional form of application (MeJ). However, it must be emphasized that this form of application involves ten times less the concentration of MeJ, which translates into a lower environmental impact and is also of special interest for the economy of the viticulturist.

Finally, do not forget that a higher nitrogen concentration in grapes and wines due to the use of MeJ treatments could lead to the higher synthesis of biogenic amines in musts and wines, which can cause adverse health troubles to the consumers.

## Figures and Tables

**Table 1:** Climatic conditions during two seasons (2019 and 2020).

	2019				2020			
	July	August	September	October	July	August	September	October
Rmean (w/m^2^)	363	352	376	375	365	356	295	271
Rainfall (mm)	1.4	22.7	147.7	39.2	4.7	0.4	5.3	8.7
Tmin (°C)	22.8	21.4	15.8	9.6	21.5	19.4	16.2	9.5
Tmax (°C)	31.6	28.4	24.3	19.8	28.7	29.1	23.1	20.0
Tmean (°C)	25.5	24.9	20.7	15.7	25.3	25.1	20.3	14.0

Abbreviations: Rmean, mean radiation; Tmin, minimum temperature; Tmax, maximum temperature; Tmean, medium temperature.

**Table 2:** Physicochemical parameters of Monastrell must at harvest.

	2019				2020			
	Control	MeJ	MeJ-ACP	*p*-Value	Control	MeJ	MeJ-ACP	*p*-Value
°Brix	23.4	23.2	23.5	ns	26.3	25.6	24.9	ns
pH	3.8	3.9	3.8	ns	4.1	4.1	4.1	ns
Total acidity (g/L) ^a^	2.8	3.2	2.9	ns	2.3b	2.7a	2.4b	*
Tartaric acid (g/L)	3.9	3.8	3.9	ns	4.5	4.4	4.2	ns
Malic acid (g/L)	1.4	1.7	1.4	ns	1.4b	1.9a	1.4b	***

^a^ Total acidity expressed as g/L tartaric acid. For each parameter and season, different letters indicate significant differences between treatments according to Duncan test (*p* > 95%). Statistically significant differences a * *p* ≤ 0.05, *** *p* ≤ 0.001; ns: not significant according to Duncan test.

**Table 3:** Nitrogen composition in Monastrell musts from non-treated and treated grapes with MeJ and MeJ-ACP (expressed as mg N/L).

	2019			2020			Multifactorial Analysis		
	Control	MeJ	MeJ-ACP	Control	MeJ	MeJ-ACP	T	S	TxS
Asp	27.6c	38.7a	33.3b	10.2	11.3	10.7	***	***	***
Glu	33.3b	43.4a	45.4a	13.1c	21.8a	16.6b	***	***	***
Asn + Ser	42.3c	106.7a	77.5b	91.0b	114.1a	114.1a	***	***	***
Gln	53.6c	148.3a	127.7b	198.5b	280.9a	213.0b	***	***	**
His	13.1c	46.3a	32.0b	69.7b	95.1a	75.3b	***	***	ns
Gly	6.0c	10.3a	7.5b	12.3	13.8	13.8	***	***	**
Thr	14.9c	42.4a	27.7b	43.1	47.9	44.4	***	***	***
β-Ala	10.5c	30.3a	19.7b	28.9b	39.6a	37.7a	***	***	*
Arg + GABA	42.5c	275.4a	188.4b	213.8c	354.3a	301.5b	***	***	**
α-Ala	21.5b	27.6a	20.3b	23.8c	30.7b	130.3a	***	***	***
Pro	199.3c	564.1a	335.0b	345.9b	449.6a	436.2a	***	***	***
NH_4_^+^	14.9c	33.0a	23.9b	21.6b	27.3a	25.1a	***	ns	***
Tyr	7.0b	11.9a	9.2b	26.1b	37.8a	37.0a	***	***	**
Val	8.9c	20.4a	14.6b	33.6	34.4	37.1	***	***	***
Met	2.8b	4.1a	3.4b	4.8b	5.5ab	6.3a	**	***	*
Cys	10.4a	7.7ab	6.8b	13.2a	9.9b	9.0b	***	**	ns
Iso	6.5c	11.0a	8.4b	15.1b	15.0b	17.5a	***	***	***
Leu	8.1c	16.6a	12.1b	24.8	25.1	27.2	***	***	***
Trp	20.3c	47.8a	35.2b	59.3b	80.1a	54.4b	***	***	**
Phe	5.3c	13.2a	9.8b	17.3	17.2	18.9	***	***	**
Orn	2.1c	2.9a	2.6b	2.6b	3.3a	2.1c	***	**	***
Lys	2.6c	4.4a	3.2b	3.4b	4.2a	2.8c	***	ns	***
Pro/Arg	4.7a	2.1b	1.8b	1.6a	1.3b	1.5ab	***	***	***
Totals	553.6c	1506.7a	1043.6b	1271.9b	1718.8a	1631.3a	***	***	***
Totals-Pro	354.3c	942.5a	708.6b	926.0b	1269.2a	1195.1a	***	***	**

For each parameter and season, different letters indicate significant differences between treatments according to Duncan Test (*p >* 95%). Statistically significant differences a * p ≤ 0.05, ** p ≤ 0.01 and *** p ≤ 0.001; ns: not significant according to Duncan Test. Abbreviations: T, treatment; S, Season; see Supplementary Materials Table S1 regarding amino acid names.

**Table 4:** Nitrogen composition in Monastrell wines from non-treated and treated grapes with MeJ and MeJ-ACP at the end of alcoholic fermentation (expressed as mg N/L).

	2019			2020			Multifactorial Analysis		
	Control	MeJ	MeJ-ACP	Control	MeJ	MeJ-ACP	T	S	TxS
Asp	2.1b	4.0a	3.6a	3.3c	5.2a	4.1b	***	***	ns
Glu	4.1b	9.8a	8.3a	9.4b	18.8a	12.4b	***	***	ns
Asn + Ser	1.3c	5.9a	3.8b	6.4b	10.7a	8.7ab	***	***	ns
Gln	2.2b	7.1a	5.8a	5.7b	8.4a	7.3a	***	***	ns
His	3.4b	4.8a	4.6a	5.6	7.9	5.9	**	***	ns
Gly	4.6b	6.6a	6.1a	7.7b	11.1a	9.3ab	***	***	ns
Thr	3.4b	3.8a	3.8a	4.2b	5.2a	4.4b	***	***	*
β-Ala	2.4b	2.8a	2.8a	3.6	4.5	3.6	ns	***	ns
Arg	4.3c	7.4a	6.3b	10.8b	21.7a	15.3b	***	***	**
GABA	4.7c	10.8a	8.8b	15.4b	27.2a	19.8b	***	***	ns
α-Ala	2.2c	4.3a	3.4b	5.5b	9.9a	7.2b	***	***	*
Pro	102.7b	466.0a	358.2a	564.9b	1109.8a	893.1a	***	***	ns
NH_4_^+^	2.5	2.7	2.9	4.1b	8.5a	5.6b	***	***	***
Tyr	3.2b	4.7a	4.8a	5.4b	9.3a	6.0b	***	***	***
Val	2.6	2.9	2.7	2.9b	3.8a	3.3b	**	***	ns
Met	2.7	3.1	3.1	3.1	3.7	3.3	*	*	ns
Cys	3.2	3.2	3.3	4.6	4.2	4.0	ns	***	ns
Iso	3.6b	4.4a	4.1ab	4.7	5.5	4.7	*	***	ns
Leu	3.9b	4.9a	4.8a	5.3b	7.6a	5.7b	***	***	**
Trp	4.0b	4.8a	4.7a	5.0c	6.5a	5.8b	***	***	*
Phe	2.7b	3.9a	3.7a	3.7b	6.1a	4.2b	***	***	**
Orn	2.0b	2.5a	2.3a	3.1b	4.6a	3.5b	***	***	**
Lys	4.3b	7.6a	6.2ab	8.6b	16.0a	9.3b	**	***	ns
Totals	171.9b	578.0a	458.0a	693.0b	1316.2a	1046.7a	***	***	ns
Totals-Pro	69.2c	112.0a	99.8b	128.1b	206.3a	153.6b	***	***	*

For each parameter and season, different letters indicate significant differences between treatments according to Duncan test (*p* > 95%*).* Statistically significant differences a * p ≤ 0.05, ** p ≤ 0.01 and *** p ≤ 0.001; ns: not significant according to Duncan test. Abbreviations: T, treatment; S, season.

**Table 5:** Standardized coefficients of the discriminant functions.

	Function 1	Function 2
Asp	−0.52	0.72
Glu	−1.89	−5.44
Asn + Ser	12.34	23.68
Gln	11.45	−23.41
His	1.64	−13.10
Gly	1.10	1.28
Thr	−10.58	−8.37
β-Ala	−9.93	−7.23
Arg + GABA	−4.09	15.95
α-Ala	−1.95	1.43
Pro	19.58	−11.38
NH_4_^+^	5.29	−3.83
Tyr	6.25	−2.33
Val	2.33	0.13
Met	−1.53	1.40
Cys	−0.29	2.28
Iso	−15.69	−1.50
Leu	−3.57	10.34
Trp	−2.56	22.07
Phe	6.78	−11.34
Orn	3.60	2.13
Lys	5.50	1.16

## Data Availability

Data is contained within the article.

## Supplementary Materials

The following are available online at https://www.mdpi.com/article/10.3390/biom11111631/s1, Table S1: Abbreviations used in Table 3, Table S2: Percentage of variance attributable to treatment, season and interaction of the variables of each amino acid concentration in Monastrell musts. and Table S3: Percentage of variance attributable to treatment, season and interaction of the variables of each amino acid concentration in Monastrell wines.

## References

[1] Bell S.-J., Henschke P.A. (2005). Implications of nitrogen nutrition for grapes, fermentation and wine. Aust. J. Grape Wine Res..

[2] Garde-Cerdán T., Ancín-Azpilicueta C. (2006). Review of quality factors on wine ageing in oak barrels. Trends Food Sci. Technol..

[3] Garde-Cerdán T., Martínez-Gil A.M., Lorenzo C., Lara J.F., Pardo F., Salinas M.R. (2011). Implications of nitrogen compounds during alcoholic fermentation from some grape varieties at different maturation stages and cultivation systems. Food Chem..

[4] Bisson L.F., Butzke C.E. (2000). Diagnosis and rectification of stuck and sluggish fermentations. Am. J. Enol. Vitic..

[5] Smit I., Pfliehinger M., Binner A., Großmann M., Horst W.J., Löhnertz O. (2014). Nitrogen fertilisation increases biogenic amines and amino acid concentrations in Vitis vinifera var. Riesling musts and wines. J. Sci. Food Agric..

[6] ter Schure E.G., van Riel N.A., Verrips C.T. (2000). The role of ammonia metabolism in nitrogen catabolite repression in Saccharomyces cerevisiae. FEMS Microbiol. Rev..

[7] Magasanik B., Kaiser A.C. (2002). Nitrogen regulation in Saccharomyces cerevisiae. Gene.

[8] Bely M., Sablayrolles J.M., Barre P. (1990). Automatic detection of assimilable nitrogen deficiencies during alcoholic fermentation in enological conditions. J. Ferment. Bioeng..

[9] Gump B.H., Zoecklein B.W., Fugelsang K.C., Whiton R.S. (2002). Comparison of analytical methods for prediction of prefermentation nutritional status of grape juice. Am. J. Enol. Vitic..

[10] Huang Z., Ough C.S. (1989). Effect of vineyard locations, varieties, and rootstocks on the juice amino acid composition of several cultivars. Am. J. Enol. Vitic..

[11] Gutiérrez-Gamboa G., Mateluna-Cuadra R., Díaz-Gálvez I., Mejía N., Verdugo-Vásquez N. (2021). Methyl Jasmonate Applications in Viticulture: A Tool to Increase the Content of Flavonoids and Stilbenes in Grapes and Wines. Horticulturae.

[12] Franceschi V.R., Grimes H. (1991). Induction of soybean vegetative storage proteins and anthocyanins by low-level atmospheric methyl jasmonate. Proc. Natl. Acad. Sci. USA.

[13] Saniewski M., Miyamoto K., Ueda J. (1998). Methyl Jasmonate Induces Gums and Stimulates Anthocyanin Accumulation in Peach Shoots. J. Plant Growth Regul..

[14] Kondo S., Tsukada N., Niimi Y., Seto H. (2001). Interactions between Jasmonates and Abscisic Acid in Apple Fruit, and Stimulative Effect of Jasmonates on Anthocyanin Accumulation. J. Jpn. Soc. Hortic. Sci..

[15] Sayyari M., Babalar M., Kalantari S., Martínez-Romero D., Guillén F., Serrano M., Valero D. (2011). Vapour treatments with methyl salicylate or methyl jasmonate alleviated chilling injury and enhanced antioxidant potential during postharvest storage of pomegranates. Food Chem..

[16] Huang X., Li J., Shang H., Meng X. (2015). Effect of methyl jasmonate on the anthocyanin content and antioxidant activity of blueberries during cold storage. J. Sci. Food Agric..

[17] Flores G., Blanch G.P., del Castillo M.L.R. (2015). Postharvest treatment with (−) and (+)-methyl jasmonate stimulates anthocyanin accumulation in grapes. LWT Food Technol..

[18] Pérez A.G., Sanz C., Olías R., Ríos J.J., Olías J.M., Kader A.A. (1997). Effect of modified atmosphere packaging on strawberry quality during shelf-life. Proceedings of the Fruits Other than Apples and Pears.

[19] Larrondo F., Gaudillère J.P., Krisa S., Decendi A., Deffieux G., Mérillon J.M. (2003). Airborne methyl jasmonate induces stilbene accumulation in leaves and berries of grapevine plants. Am. J. Enol. Vitic..

[20] Garde-Cerdán T., Portu J., López R., Santamaría P. (2016). Effect of methyl jasmonate application to grapevine leaves on grape amino acid content. Food Chem..

[21] Gutiérrez-Gamboa G., Garde-Cerdán T., Gonzalo-Diago A., Moreno-Simunovic Y., Martínez-Gil A.M. (2017). Effect of different foliar nitrogen applications on the must amino acids and glutathione composition in Cabernet Sauvignon vineyard. LWT Food Sci. Technol..

[22] Ruiz-García Y., Romero-Cascales I., Gil-Muñoz R., Fernández-Fernández J.I., López-Roca J.M., Gómez-Plaza E. (2012). Improving grape phenolic content and wine chromatic characteristics through the use of two different elicitors: Methyl jasmonate versus benzothiadiazole. J. Agric. Food Chem..

[23] Ruiz-García Y., Romero-Cascales I., Bautista-Ortín A.B., Gil-Muñoz R., Martínez-Cutillas A., Gómez-Plaza E. (2013). Increasing bioactive phenolic compounds in grapes: Response of six monastrell grape clones to benzothiadiazole and methyl jasmonate treatments. Am. J. Enol. Vitic..

[24] Gil-Muñoz R., Bautista-Ortín A.B., Ruiz-García Y., Fernández-Fernández J.I., Gómez-Plaza E. (2017). Improving phenolic and chromatic characteristics of Monastrell, Merlot and Syrah wines by using methyl jasmonate and benzothiadiazole. J. Int. Sci. Vigne. Vin..

[25] Portu J., López-Alfaro I., Gómez-Alonso S., López R., Garde-Cerdán T. (2015). Changes on grape phenolic composition induced by grapevine foliar applications of phenylalanine and urea. Food Chem..

[26] DeRosa M.C., Monreal C., Schnitzer M., Walsh R., Sultan Y. (2010). Nanotechnology in fertilizers. Nat. Nanotechnol..

[27] Kottegoda N., Sandaruwan C., Priyadarshana G., Siriwardhana A., Rathnayake U.A., Berugoda Arachchige D.M., Amaratunga G.A. (2017). Urea-hidroxyapatite nanohybrids for slow release of nitrogen. ACS Nano.

[28] Dimkpa C.O., White J.C., Elmer W.H., Gardea-Torresdey J. (2017). Nanoparticle and Ionic Zn Promote Nutrient Loading of Sorghum Grain under Low NPK Fertilization. J. Agric. Food Chem..

[29] White J.C., Gardea-Torresdey J. (2018). Achieving food security through the very small. Nat. Nanotechnol..

[30] Parra-Torrejón B., Ramírez-Rodríguez G.B., Giménez-Bañón M.J., Moreno-Olivares J.D., Paladines-Quezada D.F., Gil-Muñoz R., Delgado-López J.M. (2021). Nanoelicitors with prolonged retention and sustained release to produce beneficial compounds in wines. Environ. Sci. Nano.

[31] Pérez-Alvarez E.P., Ramirez-Rodriguez G., Martinez-Vidaurre J.M., Masciocchi N., Guagliardi A., Garde-Cerdán T., Delgado-López J.M. (2021). Towards a more sustainale viticulture: Foliar application of N-doped calcium phosphate nanoparticles on Tempranillo grapes. J. Sci. Food Agric..

[32] OIV (2018). Compendium of Internationals Methods of Wine and Must Analysis.

[33] Gómez-Alonso S., Hermosín-Gutiérrez I., García-Romero E. (2007). Simultaneous HPLC analysis of biogenic amines, amino acids and ammonium ion as aminoenone derivative in wine and beer samples. J. Agric. Food Chem..

[34] Portu J., López R., Baroja E., Santamaría P., Garde-Cerdán T. (2016). Improvement of grape and wine phenolic content by foliar application to grapevine of three different elicitors: Methyl jasmonate, chitosan, and yeast extract. Food Chem..

[35] Paladines-Quezada D., Moreno-Olivares J., Fernández-Fernández J., Bautista-Ortín A., Gil-Muñoz R. (2019). Influence of methyl jasmonate and benzothiadiazole on the composition of grape skin cell walls and wines. Food Chem..

[36] D’Onofrio C., Matarese F., Cuzzola A. (2018). Effect of methyl jasmonate on the aroma of Sangiovese grapes and wines. Food Chem..

[37] Oliva J., Garde-Cerdán T., Martínez-Gil A.M., Salinas M.R., Barba A. (2011). Fungicide effects on ammonium and amino acids of Monastrell grapes. Food Chem..

[38] Garde-Cerdán T., Gutiérrez-Gamboa G., Portu J., Fernández-Fernández J.I., Gil-Muñoz R. (2017). Impact of phenylalanine and urea applications to Tempranillo and Monastrell vineyards on grape amino acid content during two consecutive vintages. Food Res. Int..

[39] Gutiérrez-Gamboa G., Pérez-Álvarez E.P., Rubio-Bretón P., Garde-Cerdán T. (2019). Foliar application of methyl jasmonate to Graciano and Tempranillo vines: Effects on grape amino acid content during two consecutive vintages. Oeno One.

[40] Bouzas-Cid Y., Díaz-Losada E., Trigo-Córdoba E., Falqué E., Orriols I., Garde-Cerdán T., Mirás-Avalos J.M. (2018). Effects of irrigation over three years on the amino acidcomposition of Albariño (*Vitis vinifera* L.) musts and wines in two different terroirs. Sci. Hortic..

[41] Gutiérrez-Gamboa G., Garde-Cerdán T., Rubio-Bretón P., Pérez-Alvarez E.P. (2020). Study of must and wine amino acids composition after seaweed applications to Tempranillo blanco grapevines. Food Chem..

[42] Ruiz-García Y., Gómez-Plaza E. (2013). Elicitors: A Tool for Improving Fruit Phenolic Content. Agriculture.

[43] Gutiérrez-Gamboa G., Carrasco-Quiroz M., Martínez-Gil A.M., Pérez-Álvarez E.P., Garde-Cerdán T., Moreno-Simunovic Y. (2018). Grape and wine amino a composition from Carignan noir grapevines growing under rainfed conditions in the Maule Valley. Chile: Effects of location and rootstock. Food Res. Int..

[44] Hernández-Orte P., Ibarz M., Cacho J., Ferreira V. (2006). Addition of amino acids to grape juice of the Merlot variety: Effect on amino acid uptake and aroma generation during alcoholic fermentation. Food Chem..

[45] Landaud S., Helinck S., Bonnarme P. (2008). Formation of volatile sulfur compounds and metabolism of methionine and other sulfur compounds in fermented food. Appl. Microbiol. Biotechnol..

[46] Gutiérrez-Gamboa G., Portu J., Santamaría P., López R., Garde-Cerdán T. (2017). Effects on grape amino acid concentration through foliar application of three different elicitors. Food Res. Int..

[47] Martínez-Gil A.M., Garde-Cerdán T., Lorenzo C., Lara J.F., Pardo F., Salinas M.R. (2011). Volatile Compounds Formation in Alcoholic Fermentation from Grapes Collected at 2 Maturation Stages: Influence of Nitrogen Compounds and Grape Variety. J. Food Sci..

[48] Sánchez-Gómez R., Garde-Cerdán T., Zalacain A., Garcia R., Cabrita M., Salinas M., Sánchez-Gómez R., Garde-Cerdán T., Zalacain A., Garcia R. (2016). Vine-shoot waste aqueous extract applied as foliar fertilizer to grapevines: Effect on amino acids and fermentative volatile content. Food Chem..

[49] Garde-Cerdán T., Lorenzo C., Lara J.F., Pardo F., Ancín-Azpilicueta C., Salinas M.R. (2009). Study of the Evolution of Nitrogen Compounds during Grape Ripening. Application to Differentiate Grape Varieties and Cultivated Systems. J. Agric. Food Chem..

[50] Stines A., Grubb J., Gockowiak H., Henschke P., Høj P., van Heeswijck R. (2000). Proline and arginine accumulation in developing berries of *Vitis vinifera* L. in Australian vineyards: Influence of vine cultivar, berry maturity and tissue type. Aust. J. Grape Wine Res..

[51] Orte M.P.H., Ibarz M., Cacho J., Ferreira V. (2005). Effect of the addition of ammonium and amino acids to musts of Airen variety on aromatic composition and sensory properties of the obtained wine. Food Chem..

[52] Lonvaud-Funel A., Moreno-Arribas M.V., Bartolomé Suáldea B. (2016). Undesirable Compounds and Spoilage Microorganisms in Wine. Wine Safety, Consumer Preference, and Human Health.

[53] Lorenzo C., Bordiga M., Pérez-Álvarez E., Travaglia F., Arlorio M., Salinas M.R., Coïsson J.D., Garde-Cerdán T. (2017). Impacts of temperature, alcoholic degree and amino acids content on biogenic amines and their precursor amino acids content in red wine. Food Res. Int..

[54] Pérez-Álvarez E.P., Garde-Cerdán T., Cabrita M.J., García-Escudero E., Peregrina F. (2017). Influence on wine biogenic amine composition of modifications to soil N availability and grapevine N by cover crops. J. Sci. Food Agric..

[55] Díez L., Solopova A., Fernández-Pérez R., González M., Tenorio C., Kuipers O.P., Ruiz-Larrea F. (2017). Transcriptome analysis shows activation of the argininedeiminase pathway in Lactococcus lactis as a response to ethanol stress. Int. J. Food Microbiol..

[56] Parker M., Capone D.L., Francis I.L., Herderich M.J. (2017). Aroma Precursors in Grapes and Wine: Flavor Release during Wine Production and Consumption. J. Agric. Food Chem..

[57] Gamboa G.G., Garde-Cerdán T., Portu J., Moreno-Simunovic Y., Martínez-Gil A.M. (2017). Foliar nitrogen application in Cabernet Sauvignon vines: Effects on wine flavonoid and amino acid content. Food Res. Int..

[58] Garde-Cerdán T., López R., Portu J., González-Arenzana L., López-Alfaro I., Santamaría P. (2014). Study of the effects of proline, phenylalanine, and urea foliar application to Tempranillo vineyards on grape amino acid content. Comparison with commercial nitrogen fertilisers. Food Chem..

[59] Gamboa G.G., Portu J., López R., Santamaría P., Garde-Cerdán T. (2017). Elicitor and nitrogen applications to Garnacha, Graciano and Tempranillo vines: Effect on grape amino acid composition. J. Sci. Food Agric..

[60] Ougha C.S., Kriel A. (1985). Ammonia Concentrations of Musts of Different Grape Cultivars and Vineyards in the Stellenbosch Area. S. Afr. J. Enol. Vitic..

[61] Alcaide-Hidalgo J., Moreno-Arribas M.V., Martín-Álvarez P.J., Polo M.C. (2007). Influence of malolactic fermentation, postfermentative treatments and ageing with lees on nitrogen compounds of red wines. Food Chem..

