# Supplementary material for: Effect of Methyl Jasmonate Doped Nanoparticles on Nitrogen Composition of Monastrell Grapes and Wines
Source: Biomolecules. 2021 Nov 4;11(11):1631. doi: 10.3390/biom11111631 (PMC8615355; doi:10.3390/biom11111631)
Supplement: Supplementary file 1 [file biomolecules-11-01631-s001.zip › biomolecules-1427179-supplementary.pdf]

biomolecules

MDPI

Supplementary

Table S1. Abbreviations used in Table 3.

Asp	Aspartic acid
Glu	Glutamic acid
Asn + Ser	Asparagine+serine
Gln	Glutamine
His	Histidine
Gly	Glycine
Thr	Threonine
β-Ala	β-Alanine
Arg + GABA	Arginine+γ-aminobutyric acid
α-Ala	α-Alanine
Pro	Proline
NH_4^+	Ammonium ion
Tyr	Tyrosine
Val	Valine
Met	Methionine
Cys	Cysteine
Iso	Isoleucine
Leu	Leucine
Trp	Tryptophan
Phe	Phenylalanine
Orn	Ornithine
Lys	Lysine

Table S2. Percentage of variance attributable to treatment. season and interaction of the variables of each amino acid concentration in Monastrell musts.

	**Treatment**	**Season**	**T x S**	**Residual**
Asp	4.48	92.18	3.00	0.35
Glu	10.60	86.53	2.16	0.71
Asn + Ser	49.98	36.04	11.36	2.62
Gln	25.02	69.99	3.16	1.82
His	18.91	80.12	0.97	2.38
Gly	15.55	79.46	4.99	2.70
Thr	32.11	51.94	15.95	3.38
B-Ala	38.47	57.23	4.30	4.37
Arg + GABA	59.55	36.80	3.65	2.03
α-Ala	2.38	96.00	1.62	0.46
Pro	71.06	3.50	25.44	1.26
NH4+	78.20	0.45	21.34	6.73
Tyr	7.80	90.03	2.17	1.29
Val	5.89	89.57	4.54	1.33
Met	15.78	77.43	6.79	9.02
Cys	52.46	26.43	0.49	20.62
Iso	6.62	82.78	8.39	2.21
Leu	6.58	87.28	6.13	1.77
Trp	29.78	62.38	4.60	3.24
Phe	11.68	72.09	10.98	5.26

Orn	67.22	3.09	26.27	3.42
Lys	78.81	0.40	14.38	6.41
Pro/Arg	36.73	35.03	26.07	2.17
Totales	49.45	44.83	5.73	1.66
Totales-Pro	27.58	70.83	1.60	1.47

Table S3. Percentage of variance attributable to treatment. season and interaction of the variables of each amino acid concentration in Monastrell wines.

	**Treatment**	**Season**	**T x S**	**Residual**
Asp	66.40	26.02	2.60	4.98
Glu	42.16	43.51	4.77	9.56
Asn + Ser	32.54	62.46	0.06	4.94
Gln	59.15	25.37	5.81	9.67
His	28.95	64.58	6.47	14.94
Gly	27.67	70.28	2.05	5.48
Thr	22.68	69.78	7.55	6.21
β-Ala	14.77	78.10	7.13	18.63
Arg	22.51	70.51	6.98	3.85
GABA	23.33	73.73	2.94	5.85
Al	26.55	69.84	3.61	3.33
Pro	31.48	67.32	1.19	5.46
NH4+	19.53	63.51	16.97	5.43
Tyr	34.79	52.01	13.19	2.47
Val	41.14	49.81	9.05	15.66
Met	27.77	33.52	3.23	35.49
Cys	2.26	66.05	5.55	26.13
Iso	28.45	67.22	4.33	23.92
Leu	34.20	50.74	9.82	5.24
Trp	32.25	58.56	4.06	5.13
Phe	46.77	36.64	10.51	6.08
Orn	19.75	71.08	5.60	3.57
Lys	37.12	53.50	9.39	14.32
Totales	31.46	67.17	1.37	5.30
Totales-Pro	31.70	64.22	4.08	5.49
